# Self-Cure

**Published:** 1886-12

**Authors:** 


### SELF-CURE.

The body, to a large extent, is a machine which, when disarranged, repairs itself. Physicians tell us of the *vis medica, rix naturæ*—the power to heal inherent in nature. It is natural to get well. The body's recuperative resources are not equal to every need, but they are very great. It is because of this even that the well man tends to keep well, if he conforms to nature's laws, for the system is ever full of poison from its own waste, the disposal of which nature has provided for, better than any city has for the disposal of its deadly sewerage.

Take the case of an ordinary wound. It needs only to have its disrupted parts brought together and nature does the healing ; and even in many cases where the parts are not brought together, nature fills up the space with new flesh. So nature will mend a broken bone, on the simple condition that the adjusted parts be allowed the requisite rest.

Dyspepsia, whether induced by improper eating, the neglect of exercise, brain overwork, or care, worry and fret, will in time wholly disappear on removal of the cause and compliance with the laws of nature.

The best physicians now freely admit that typhoid patients, in the great majority of cases, would recover without a drop of medicine ; that they need medicine mainly to promote ease and comfort, and that pure air is better for them than all drugs. The same is true of some other diseases. More and more is it being admitted that, in no case, no drugs have any curative powers, but only aid nature, as the surgeon aids in the case of a badly broken limb, by removing irritating bits, spiculæ, etc., and securing the proper adjustment and fixation of the parts.

The old-time doctors greatly overdosed people—in multitudes of cases literally dosed people to death. Within less than twenty years a personal friend, called to watch with a neighbor far gone in consumption, was shown eleven different medicines, each of which she was to administer during the night, according to the varying symptoms.

It cannot be too strongly emphasized that those who observe the laws of their physical nature are likely to keep well—and even infectious diseases have little power over such persons, and would wholly disappear if all observed these laws.

